# Visualization of Anatomic Variation of the Anterior Septal Vein on Susceptibility-Weighted Imaging

**DOI:** 10.1371/journal.pone.0164221

**Published:** 2016-10-07

**Authors:** Zhengzhen Chen, Huihuang Qiao, Yu Guo, Jiance Li, Huizhong Miao, Caiyun Wen, Xindong Wen, Xiaofen Zhang, Xindong Yang, Chengchun Chen

**Affiliations:** 1 Department of Human Anatomy, Wenzhou Medical University, Wenzhou, Zhejiang, China; 2 Department of Radiology, The 2^nd^ hospital of Huangshi, Huangshi, Hubei, China; 3 Department of Radiology, the 1^st^ Affiliated Hospital of Wenzhou Medical University, Wenzhou, Zhejiang, China; University of North Carolina at Chapel Hill, UNITED STATES

## Abstract

**Background and Purpose:**

Understanding the anatomy of the anterior septal vein (ASV) is critical for minimally invasive procedures to the third ventricle and for assessing lesion size and venous drainage in the anterior cranial fossa. Accordingly, this study evaluated topographic anatomy and anatomic variation of the ASV using susceptibility-weighted imaging (SWI).

**Methods:**

Sixty volunteers were examined using a 3.0T MR system. The diameter of the ASV and distance between bilateral septal points were measured. ASVs were divided into types 1 (only drains frontal lobe) and 2 (drains both frontal lobe and head of the caudate nucleus). We evaluated the ASV-internal cerebral vein (ICV) junction based on its positional relationship with the appearance of a venous angle or a false venous angle and the foramen of Monro. Fused SW and T1-weighted images were used to observe positional relationships between the course of the ASV and the surrounding brain structures.

**Results:**

The ASV and its small tributaries were clearly visualized in 120 hemispheres (100%). The average diameter of ASVs was 1.05±0.17 mm (range 0.9–1.6 mm). The average distance between bilateral septal points was 2.23±1.03 mm (range 1.3–6.6 mm). The ASV types 1 and 2 were in 77 (64.2%) and 43 (35.8%) hemispheres, respectively. In 83 (69.2%) hemispheres, the ASV-ICV junction was situated at the venous angle and the posterior margin of the foramen of Monro. In 37 (30.8%) hemispheres, the ASV-ICV junction was situated beyond the posterior margin of the foramen of Monro. The average distance between the posteriorly located ASV-ICV junction and the posterior margin of the foramen of Monro was 6.41±3.95 mm (range 2.4–15.9 mm).

**Conclusion:**

Using SWI, the topographic anatomy and anatomic variation of the ASV were clearly demonstrated. Preoperative assessment of anatomic variation of the ASV may be advantageous for minimally invasive neurosurgical procedures.

## Introduction

As one of the subependymal veins, the chief function of the anterior septal vein (ASV) is to drain the deep white matter of the frontal lobe via deep medullary veins [1]. Various diseases have been shown to be associated with abnormalities of the deep medullary veins, such as stroke [2, 3], leukoaraiosis [4], and developmental venous anomaly [5]. Abnormalities of deep medullary veins in the frontal lobe may reflect poor reflux of the ASV. Previous studies have described the anatomy of the ASV using angiography, magnetic resonance venography (MRV), or autopsies [6–8]. However, those methods have certain disadvantages, such as invasiveness, use of radioactive materials, inefficiency, technical issues that render it difficult to distinguish anatomical variance, and inadequate resolution to visualize small tributaries of the ASV. Some scholars have suggested that the junction formed by the ASV and the internal cerebral vein (ICV) may play a significant role in minimally invasive procedures to the third ventricle [8–11]. However, there is a lack of research regarding methods of imaging the ASV and its small tributaries.

Susceptibility-weighted imaging (SWI), such as T2*-weighted angiography (SWAN, General Electric), susceptibility weighted imaging (SWI, Siemens), and venous blood oxygen-level dependent imaging (VenoBOLD, Philips) are useful and relatively novel magnetic resonance imaging (MRI) sequences that exploit susceptibility differences between the venous deoxygenated blood and the surrounding brain tissues [12–14]. Deoxyhemoglobin serves as an intrinsic contrast agent to generate the high-resolution venous images. Compared with conventional MRI sequences, SWI has higher sensitivity to detect deoxygenated hemoglobin, calcification, and iron content [12]. Increasingly many clinical applications of SWI of the brain have been reported, such as prediction of stroke severity [2, 3], dural arteriovenous fistula [15], and cerebral neoplasms [16]. Currently, SWI is also widely used in visualization of the cerebral venous system [17–19].

To our knowledge, there is a lack of available research describing the use of SWI to visualize the ASV in detail. In this study, we illustrate the topographic anatomy and anatomic variation of the ASV by application of SWI in a healthy cohort.

## Materials and Methods

### Volunteer Selection

Participants were 60 healthy volunteers (28 females and 32 males; age range 18–30 years; average age 26.1 years). None had cerebral disease or cerebral trauma. Informed consent forms were obtained from all volunteers. This study was approved by the Ethics Committee of Wenzhou Medical University.

### MR Imaging Technique

All volunteers were scanned via a 3.0 Tesla TX-series MRI scanner (Royal Philips Electronics, Amsterdam, Netherlands) with an 8-channel high-resolution brain-phased array coil. The following scan protocols were performed: (1) T1-weighted imaging (T1WI) and fluid-attenuated inversion recovery (FLAIR) sequence (repetition time [TR]/echo time [TE] = 1900 ms/20 ms, flip angle = 90°, image matrix = 256 × 141, field of view [FOV] = 230 mm, section thickness = 6 mm, gap between sections = 1 mm); (2) T2-weighted imaging (T2WI) and turbo spin-echo (TSE) sequence (TR/TE = 2100 ms/80 ms, flip angle = 90°, image matrix = 352 × 285, FOV = 230 mm, section thickness = 6 mm, gap between sections = 1 mm); (3) T2 FLAIR sequence (TR/TE = 6000 ms/123 ms, flip angle = 90°, image matrix = 268 × 143, FOV = 230 mm, section thickness = 6 mm, gap between sections = 1 mm); (4) SWI (VenoBOLD) sequence, collected via a 3D multishot fast field echo-echo planar imaging (FFE-EPI) sequence (TR/TE = shortest (21 ms)/shortest (32 ms), flip angle = 10°, image matrix = 316 × 362, FOV = 220 mm, voxel size = 0.7 × 0.5 × 1 mm^3^ (reconstruction matrix 1024, reconstruction voxel size 0.21 mm), and overcontiguous slice).

### Image Processing

Images were postprocessed using the Extended MR Workspace release 2.6.3.4 workstation (Philips Medical Systems, Netherlands) and reconstructed using minimum intensity projections (mIPs) technique with section thickness of 20 mm and section gap of -19 mm in the transverse plane. We fused the SW images and the corresponding T1-weighted images using Adobe Photoshop CC 2015 (AdobeSystems, USA), to observe the positional relationship between the course of the ASV and the surrounding brain structures. The main image registration steps were as follows: First, we ensured the reconstructed SW images and T1-weighted images represented the same section. Both T1WI and SWI had the same scanning location standard. Both transverse sections of the two sequences were parallel to the anterior commissure-posterior commissure (AC-PC) line. Therefore, we ensured the same sections of the two sequences were represented using the option “link image position” in the workstation. To improve accuracy, we also calculated whether the chosen sections of the two sequences were the same on the basis of the section thickness and section gap. Second, we rendered the SW images and T1-weighted images at the same size, using Adobe Photoshop CC. Third, we dragged the T1-weighted image over the SW image, such that they fully overlapped, after which we altered the T1-weighted image layer using the options “multiply” and “cancel R” under “blending options.” Brightness, contrast, and color gradation were adjusted as needed to obtain the final image.

### Evaluation

**Fig 1:**
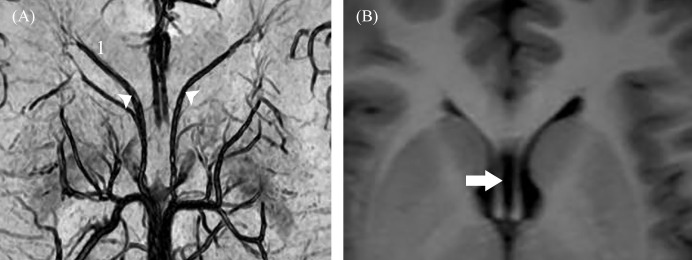
**A, Transverse SW image showing the anterior septal vein and septal points**. The distance between bilateral septal points (white arrowhead) is 6.6 mm. **B, Transverse T1-weighted image from the same volunteer showing a wider cavum septi pellucidi.** (1, anterior septal vein; white arrow, cavum septi pellucidi).

A senior neuroradiologist (J.C.L) and a specialist in sectional anatomy (C.C.C) consensually evaluated the anatomic variations of the bilateral ASVs in the transverse reconstructed SW images. The diameter of the ASV was measured adjacent to the ASV-ICV junction, using the workstation measuring scale. The septal points (Fig 1A) were determined according to the definition of Zimmer *et al*. [7], who proposed the septal point was the cusp of the angle formed by the most anterior and inferior tributary of the ASV. The septal point demarcated the septum pellucidum (Fig 1B).

**Fig 2:**
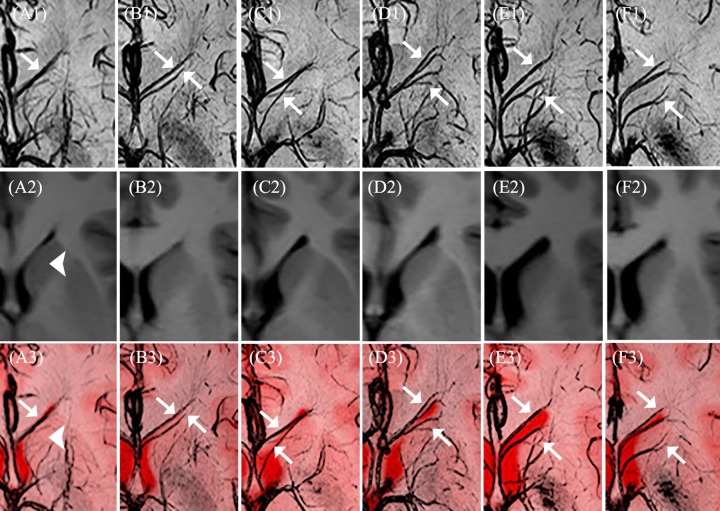
Different types of anterior septal veins. SW images (A1-F1) and the corresponding T1-weighted images (A2-F2) are fused to show the anterior septal veins and their drainage (A3-F3): A1-C1 and A3-C3 represent type 1, and D1-F1 and D3-F3 represent type 2. (arrow, anterior septal vein; arrowhead, caudate nucleus).

From our observations, even though ASVs and their tributaries varied, we divided them into two types according to their drainage: type 1, which only drained the frontal lobe region (Fig 2A–2C); type 2, which drained both the frontal lobe and the partial head of the caudate nucleus (Fig 2D–2F).

We described the variations of the ASV-ICV junction on the basis of the proposals of Türe *et al*. [9]. They noted that the position of the ASV-ICV junction had some variations. Four types were suggested on the basis of their relationship with the appearance of a venous angle or a false angle and the foramen of Monro (a venous angle was defined as the U-shaped junction formed by the thalamostriate vein (TSV) and the ICV adjacent to the posterior margin of the foramen of Monro, while a false venous angle was defined as the U-shaped junction formed by the TSV and ICV situated beyond the posterior margin of the foramen of Monro).

**Fig 3:**
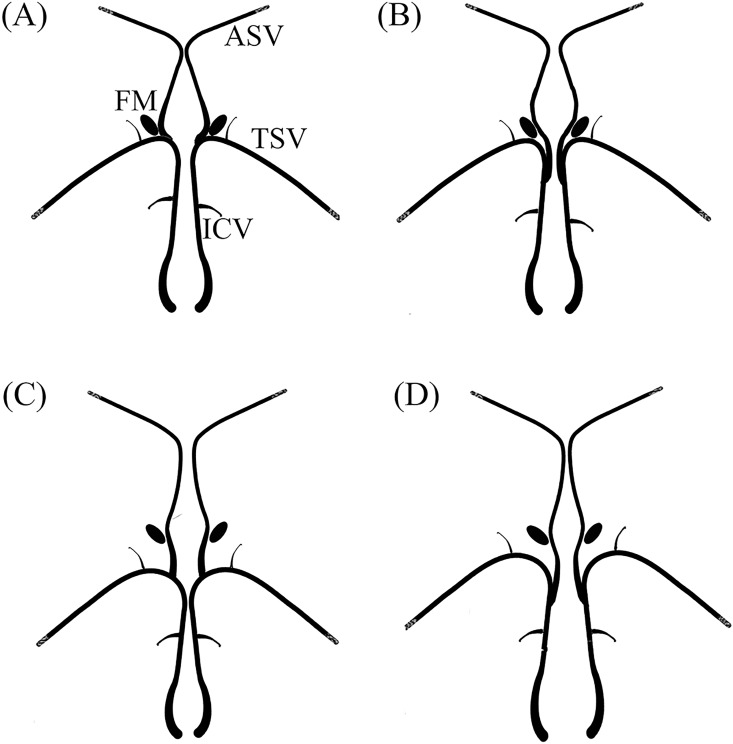
Schematic diagrams showing variations of the ASV-ICV junction. A, Type IA; B, Type IB; C, Type IIA; D, type IIB. (ASV, anterior septal veins; TSV, thalamostriate vein; FM, foramen of Monro; ICV, internal cerebral vein).

In type IA, the ASV-ICV junction was situated at the venous angle adjacent to the posterior margin of the foramen of Monro (Fig 3A). In the remaining types, the ASV-ICV junction was situated beyond the posterior margin of the foramen of Monro, with one of three different locations:

In type IB, the ASV joined the main trunk of the ICV beyond both the venous angle and the foramen of Monro (Fig 3B).

In type IIA, the ASV joined the false venous angle, located beyond the foramen of Monro (Fig 3C).

In type IIB, the ASV joined the main trunk of the ICV far beyond both the false venous angle and the foramen of Monro (Fig 3D).

### Statistical Analysis

A χ^2^ test was performed to determine significant differences in the types of ASV and the ASV-ICV junction between bilateral hemispheres. Statistical significance was set at an alpha value of 0.05. All statistical analyses were completed using SPSS version 19.0 (IBMSPSS, Chicago, IL, USA).

## Results

**Fig 4:**
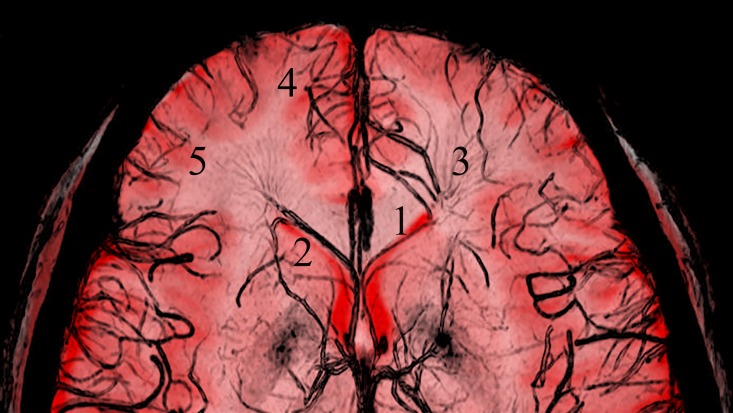
The fused image formed from T1-weighted and SW images. The positional relationships between the anterior septal vein and the surrounding brain structures are clearly observed. (1, anterior septal vein; 2, anterior horn of lateral ventricle; 3, deep medullary veins; 4, superior frontal gyrus; 5, middle frontal gyrus).

The ASV and its small tributaries were clearly visualized as hypointense linear structures in all 120 hemispheres when visualized using SWI in the transverse plane. From observation of fused images (Fig 4), the ASV ran posteromedially from the tip of the anterior horn of lateral ventricle, then turned backward along the septum pellucidum and column of the fornix. Then, it joined the ICV after passing above the foramen of Monro. The ASV received multiple deep medullary veins from the deep white matter of the superior and middle frontal gyri. Those deep medullary veins distributed as wedge-shaped patterns along the anterior horn of the lateral ventricle and some of them anastomosed with the superficial medullary veins.

The average diameter of the ASV was 1.05±0.17 mm (range 0.9–1.6 mm). The average distance between the bilateral septal points was 2.23±1.03 mm (range 1.3–6.6 mm; data in S1 Table). In 77 (64.2%) hemispheres, the ASV only drained the frontal lobe (type 1). In 43 (35.8%) hemispheres, the ASV drained both the frontal lobe and the partial head of the caudate nucleus (type 2). Data regarding the types of ASV are shown in Table 1 and S1 Table. No significance difference was found between left and right hemispheres regarding the types of ASVs. Additionally, we found that 15% of 60 volunteers had bilateral hemispheres with different types of ASVs.

**Table 1:** ASV types in 60 volunteers (120 hemispheres).

	Number (%)			
Type of ASV	Total	Left	right	*P*-value
Type 1	77(64.2)	34 (56.7)	43 (71.7)	0.09
Type 2	43 (35.8)	26 (43.3)	17 (28.3)	

ASV, anterior septal vein.

**Fig 5:**
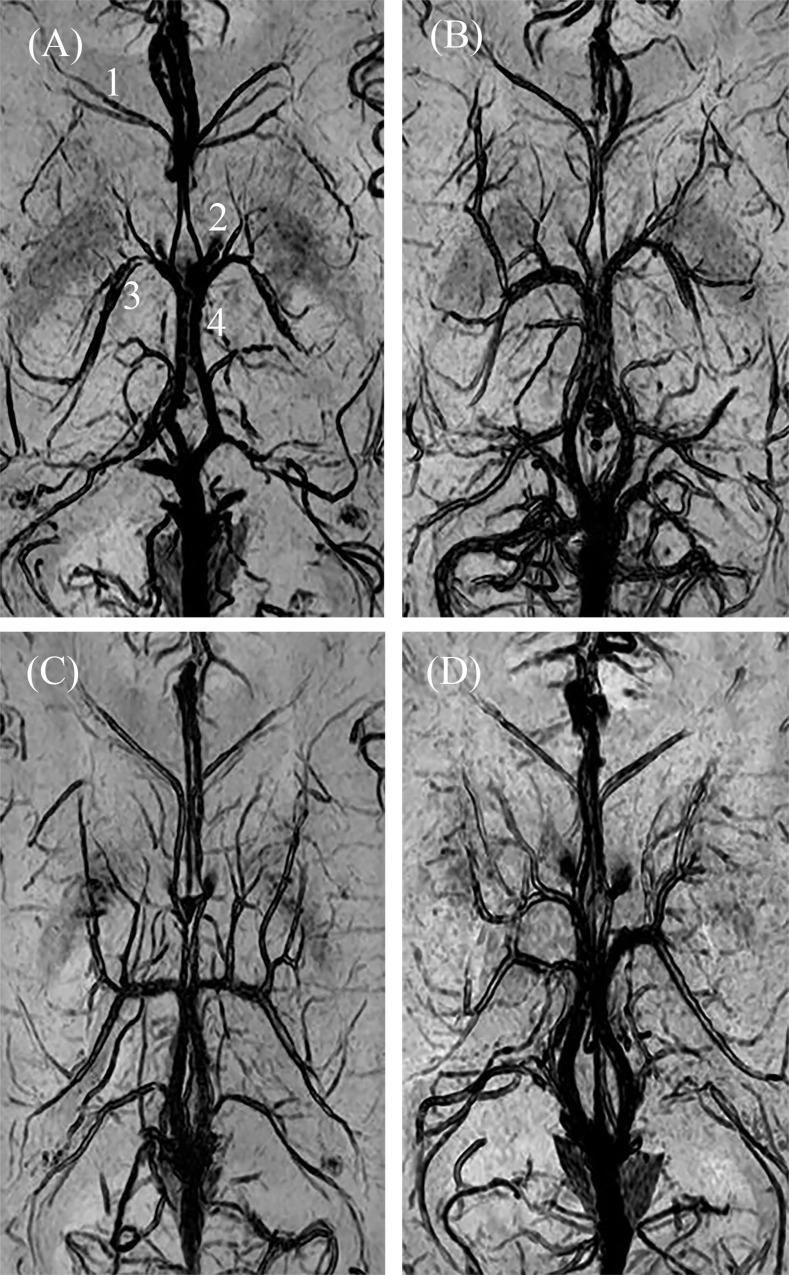
Different types of ASV-ICV junction on SWI. A, Type IA; B, Type IB (left) and type IIB (right); C, Type IIA; D, Type IIB (left) and type IIA (right). (1, anterior septal vein (ASV); 2, foramen of Monro; 3, thalamostriate vein; 4, internal cerebral vein (ICV)).

In 83 (69.2%) hemispheres, the ASV-ICV junction was situated at the venous angle (type IA). In 37 (30.8%) hemispheres, the ASV-ICV junction was situated beyond the posterior margin of the foramen of Monro (types IB, IIA, IIB). Table 2 summarizes the types of ASV-ICV junction (detailed data in S1 Table). Examples of these types are shown in Fig 5. There were no significance differences between left and right hemispheres in terms of the variations of the ASV-ICV junction.

**Table 2:** Different types of ASV-ICV junction in 60 volunteers (120 hemispheres).

	Distance from FM (mm)		Type of	Number (%)		
Type	Average	Range	venous angle	Left	Right	*P* value
IA	0	0	Venous angle	41 (68.3)	42 (70.0)	0.67
IB	4.23±0.90	3.3–5.1	Venous angle	2 (3.3)	1 (1.7)	
IIA	6.62±4.14	2.4–15.9	False venous angle	10 (16.7)	13 (21.7)	
IIB	6.55±4.09	4–13.5	False venous angle	7 (11.7)	4 (6.6)	

ASV, anterior septal vein; ICV, internal cerebral vein; FM, foramen of Monro.

In types IB, IIA, and IIB, the average distance between the ASV-ICV junction and the foramen of Monro was 6.41±3.95 mm (range 2.4–15.9 mm). The incidence of type IIA (19.2%) was higher than types IB (2.5%) and IIB (9.1%).

## Discussion

### The Advantage of SWI for Visualization of the ASV and Its Tributaries

In our study, 3.0T MRI was adopted to clearly visualize topographic anatomy and anatomic variation of the ASV using SWI (Fig A in S1 File). The ASV has a crucial role in confirming a space-occupying lesion in the anterior cranial fossa [7]. Meanwhile, the subependymal tributaries of the ASV have utility in evaluating the locations of the margins of the anterior horn of the lateral ventricle as it courses along the frontal anterior horn of the lateral ventricle. From our measurements, its average diameter was 1.05±0.17 mm (range 0.9–1.6 mm) which is similar to the values reported in an anatomical dissection study (mean value 1.02 mm, range 0.8–1.5 mm) [9]. Thus far, the gold standard to measure venous diameter is via digital subtraction angiography (DSA) in the living body. Even though Xia *et al*. [19] found that the venous diameter measurement by SWI was larger than that by DSA, there was a significant linear correlation between diameters measured by the two imaging modalities. Reichenbach *et al*. [13] pointed out that SWI suggested a diameter of approximately 1 mm for cerebral veins, but also showed cerebral veins with diameters of less than 1 mm. Our study also demonstrated that it is possible to observe the ASV and its tiny tributaries, which were less than 1 mm in diameter, on SWI with optimized sequence-parameter settings.

From observation of the fused T1WI and SWI images (Fig 4), we found that ASV was the main channel that drains the deep white matter of the frontal lobe, especially the superior and middle frontal gyri, via deep medullary veins. The directions of deep medullary veins were radial along the anterior horn of the lateral ventricle. This is consistent with a previous angiography study in the coronal plane [20]. One limitation of SWI is a loss of position information [13]; the corresponding T1-weighted images address this limitation. Our study is the first to show the normal deep medullary veins, by using the combination of SW images and T1-weighted images to analyze the positional relationship between the venous course and the surrounding brain structures. This method is easy to apply, but it requires the SW images and T1-weighted images are of the same section and have the same size. The use of link image position in the workstation software and the calculation of the section thickness and section gap for the respective images ensured that they were of the same section. It is worth noting that the two sequences had different section thickness and section gap, which led some of the SW images to have no corresponding T1-weighted image (Figs A and B in S1 File). Even though Adobe Photoshop is not specialist medical software, it is a professional image processing and graphics software package that assured the sizes of the two images were the same and that the images completely overlapped. Adobe Photoshop has been used in prior studies to fuse images to recognize the anatomical location of lesions, as well as to analyze cells and their immunologically marked intracellular constituents [21, 22].

### The Function of the ASV-ICV Junction in Surgical Exposure

Surgery of the third ventricle is challenging, even for experienced neurosurgeons. Because this region is deep and surrounded by vital neural and vascular structures, neurosurgeons are faced with a difficult problem, namely to minimize surgical injury and postoperative complications. Endoscopic and microsurgical approaches to the third ventricle have their own advantages and disadvantages [9, 23–25]. The transcallosal transforaminal approach is a safe anatomical approach with a relatively easy, direct path by which to remove lesions in the third ventricular region [9, 25, 26]. This approach takes advantage of the foramen of Monro, which is the only natural opening for surgical exploration between the lateral ventricle and the third ventricle, particularly when the foramen of Monro is dilated by lesions. However, when the foramen of Monro is not dilated by lesions, the position of the ASV–ICV junction relative to the foramen of Monro plays an important role in this approach. When the ASV-ICV junction is situated posteriorly, beyond the posterior margin of the foramen of Monro, this positioning allows enlargement of the foramen of Monro posteriorly along the choroidal fissure, as far as the junction. The choroidal fissure is the thinnest structure with no neuronal tissue in the wall of the lateral ventricle. Thus, it provides relatively open access to the third ventricle while minimizing injury to vital neural or vascular structures [25, 26]. From our observations, the position of the foramen of Monro was clear, and the distance between the foramen of Monro and the ASV-ICV junction could be accurately measured using SWI. In this study, the distance from the posterior margin of the foramen of Monro to the posteriorly located ASV-ICV junction ranged from 2.4–15.9 mm (mean 6.41±3.95 mm), which is slightly wider than in previous reports (e.g., 3–13 mm in an anatomic study [9] and 2.5–14.1 mm in a study using MRV [8]). The differences may be due to different methods and sample sizes in these prior studies and our study. The incidence of posterior location of the ASV-ICV junction is reported to vary from 19.5% to 47.5% [8–10, 27]. In our study, this location occurred in 30.8% of 120 hemispheres, which is within the ranges reported previously. From our observations, type IIA appeared with higher incidence (19.2%) than type IB (2.5%) or IIB (9.1%), similarly to Türe *et al*. [9]. There were no significance differences in variations of the ASV-ICV junction between bilateral hemispheres. Assessment of the ASV-ICV junction to the third ventricle before surgery not only allows one to take advantage of the particular anatomical structure, but also to avoid injury to the vital neural and vascular structures, which can lead to severe postoperative complications. Some neurosurgeons have taken into consideration a posteriorly located ASV-ICV junction when evaluating resection possibilities with respect to the choroidal fissure [26, 28].

### The Septal Point and Variations in ASV

In our study, the distance between the bilateral septal points ranged from 1.3–6.6 mm. However, Zimmer *et al*. [7] noted that the distance between the septal points indicated a 2–3 mm normal breadth of the septum pellucidum. Separation of these points could occur due to cavum septi pellucidi, metastasis in the septum, or tumor. Our findings suggest that SWI can visualize bilateral septal points in vivo. This could provide useful information for assessment of cavum septi pellucidi, which is a significant factor in the transcallosal interforniceal approach [29].

From our study, ASVs were divided into two types according their drainage (Fig 2). The incidences of types 1 and 2 were 64.2% and 35.8%, respectively. This suggests that most ASVs only drain the frontal lobe. There were no significance differences between left and right hemispheres with respect to the types of ASV. Some deep medullary veins anastomosed with the superficial medullary veins, which may explain why occlusion of the ASV has no clinical consequences [11, 26]. Nevertheless, injury to the ASV is still undesirable. First, injury to the ASV causes bleeding, which is detrimental to surgery of the third ventricle [11, 30]. Second, injury to a type 2 ASV, which drains the partial head of the caudate nucleus, may present a risk of caudate nucleus infarction. Further research is needed to support these suggestions. Few studies have focused on the anatomy of ASVs or their symmetry. Here, we found that 15% of 60 volunteers had bilateral hemispheres with different types of ASVs. We term this phenomenon as asymmetric ASVs. In Roth *et al*. [6], the incidence of asymmetric ASVs was 50% in 10 cadaver brains. The relative sample sizes and study methods likely underlie this difference in incidences. Careful preoperative evaluation of asymmetric ASVs is necessary to define a safe area so as to protect the contralateral ASV in septostomy [6].

## Conclusions

Using SWI, topographic anatomy and anatomic variations in the ASV were clearly demonstrated, including the diameter, course, and drainage of the ASV, septal points, and the ASV-ICV junction. Preoperative assessment of the septal points and the ASV-ICV junction may allow the neurosurgeon to utilize natural anatomic advantages during minimally invasive surgery of the third ventricular region. The fused images allow observation of the positional relationship between the ASV and the surrounding cerebral structures.

## Supporting Information

S1 File The SW and the T1-weighted images from one volunteer. Fig A. The SW images from one volunteer showing the anterior septal veins and their drainage. (white arrow, anterior septal vein). Fig B. The SW and the corresponding T1-weighted images from one volunteer. (1, 3, 5, 7, 9: SW image; 2, 4, 6, 8, 10: T1-weighted image).
(PDF)

S1 Table The SWI data of 60 volunteers. (DOCX)
