# Supplementary material for: Visualization of Anatomic Variation of the Anterior Septal Vein on Susceptibility-Weighted Imaging
Source: PLoS One. 2016 Oct 7;11(10):e0164221. doi: 10.1371/journal.pone.0164221 (PMC5055311; doi:10.1371/journal.pone.0164221)
Supplement: S1 File — Fig A. The SW images from one volunteer showing the anterior septal veins and their drainage. (white arrow, anterior septal vein). Fig B. The SW and the corresponding T1-weighted images from one volunteer. (1, 3, 5, 7, 9: SW image; 2, 4, 6, 8, 10: T1-weighted image). (PDF) [file pone.0164221.s001.pdf]

Visualization of Anatomic Variation of the Anterior Septal Vein on Susceptibility-Weighted Imaging

S1 File. The SW and the T1-weighted images from one volunteer.

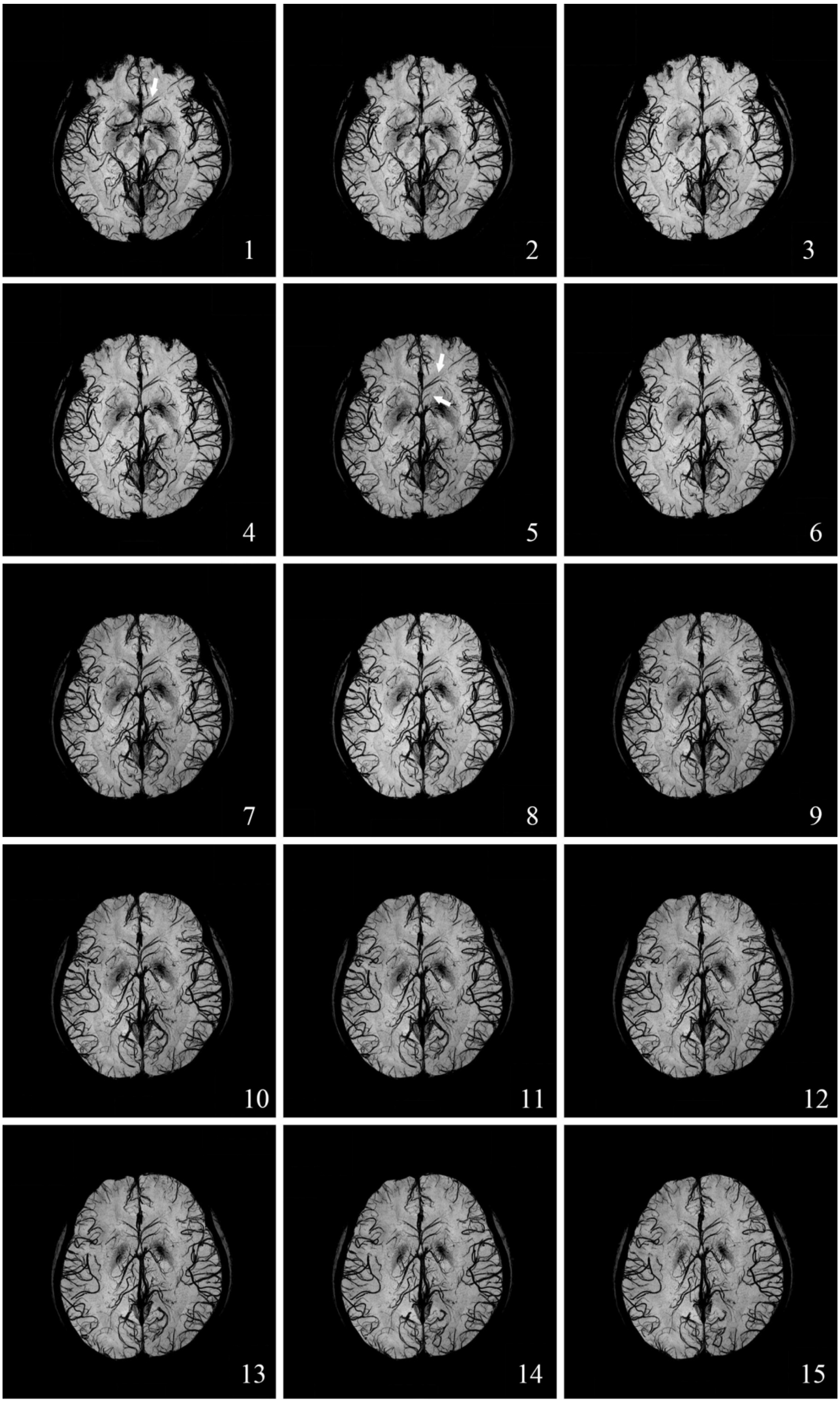

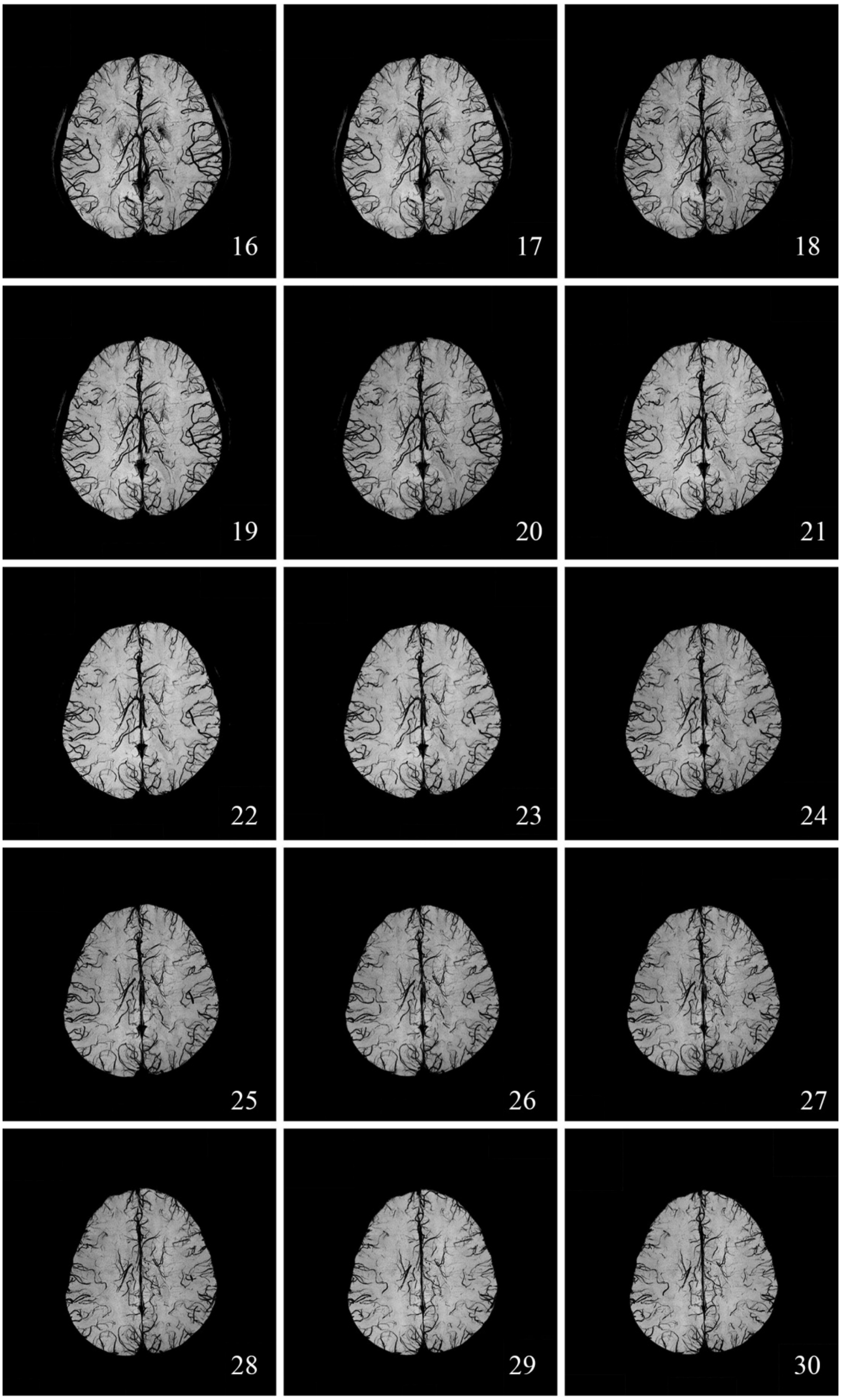

Figure A. The SW images from one volunteer showing the anterior septal veins and their drainage. (white arrow, anterior septal vein).

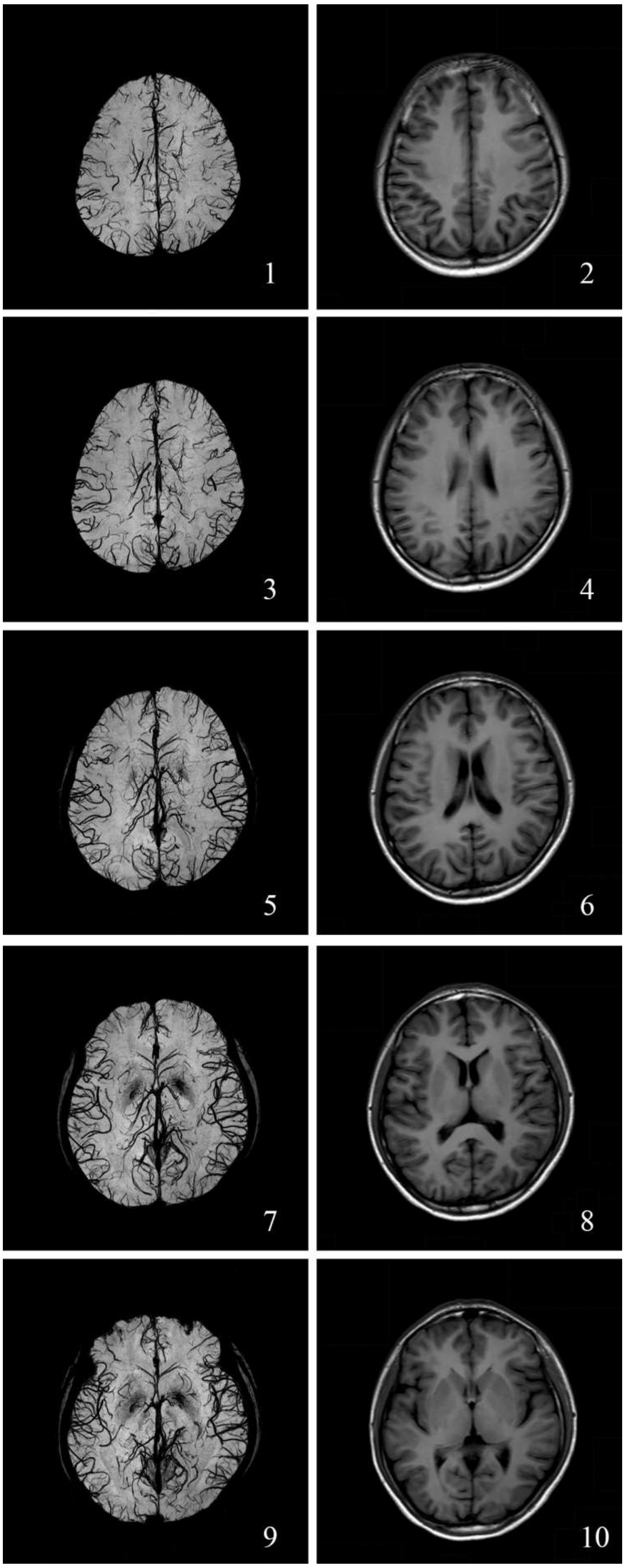

Figure B. The SW and the corresponding T1-weighted images from one volunteer. (1, 3, 5, 7, 9: SW image; 2, 4, 6, 8, 10: T1-weighted image).
