# Supplementary material for: Visualization of Anatomic Variation of the Anterior Septal Vein on Susceptibility-Weighted Imaging
Source: PLoS One. 2016 Oct 7;11(10):e0164221. doi: 10.1371/journal.pone.0164221 (PMC5055311; doi:10.1371/journal.pone.0164221)
Supplement: S1 Table — (DOCX) [file pone.0164221.s002.docx]

S1 Table. The SWI data of 60 volunteers.

| NO. | Caliber of ASV | | Distance between SPs | Types of ASV | |  | Types of ASV-ICV Junction | |  | Distance between AS-ICV junction and FM | |
| --- | --- | --- | --- | --- | --- | --- | --- | --- | --- | --- | --- |
|  | L | R |  | L | R |  | L | R |  | L | R |
| 1 | 1.2 | 1.1 | 3.3 | 1 | 1 |  | IIB | IIA |  | 12.5 | 5.5 |
| 2 | 1 | 1.1 | 2.1 | 2 | 2 |  | IA | IA |  | 0 | 0 |
| 3 | 1.1 | 0.9 | 2.2 | 2 | 2 |  | IA | IA |  | 0 | 0 |
| 4 | 1.2 | 1 | 2.1 | 1 | 1 |  | IB | IIA |  | 5.1 | 3.7 |
| 5 | 1 | 1 | 1.7 | 1 | 1 |  | IA | IA |  | 0 | 0 |
| 6 | 1.3 | 1 | 2 | 1 | 1 |  | IA | IA |  | 0 | 0 |
| 7 | 1.1 | 0.9 | 2.5 | 2 | 2 |  | IA | IA |  | 0 | 0 |
| 8 | 1 | 1.1 | 2.7 | 1 | 1 |  | IA | IA |  | 0 | 0 |
| 9 | 1.1 | 0.9 | 3.3 | 1 | 1 |  | IA | IA |  | 0 | 0 |
| 10 | 1 | 1 | 1.3 | 2 | 1 |  | IIB | IA |  | 4 | 0 |
| 11 | 1.1 | 1.1 | 1.8 | 1 | 1 |  | IB | IA |  | 4.3 | 0 |
| 12 | 1 | 0.9 | 1.4 | 2 | 1 |  | IA | IA |  | 0 | 0 |
| 13 | 1 | 1 | 1.5 | 1 | 2 |  | IIB | IA |  | 4 | 0 |
| 14 | 0.9 | 1 | 2.3 | 1 | 1 |  | IA | IA |  | 0 | 0 |
| 15 | 1 | 0.9 | 6.6 | 1 | 2 |  | IA | IA |  | 0 | 0 |
| 16 | 1 | 1.1 | 3.6 | 1 | 1 |  | IIB | IA |  | 4 | 0 |
| 17 | 1 | 1.5 | 1.8 | 2 | 1 |  | IA | IIA |  | 0 | 2.8 |
| 18 | 1.2 | 0.9 | 2.7 | 1 | 1 |  | IA | IA |  | 0 | 0 |
| 19 | 1 | 1.2 | 1.3 | 2 | 1 |  | IA | IA |  | 0 | 0 |
| 20 | 0.9 | 0.9 | 2.2 | 2 | 1 |  | IA | IA |  | 0 | 0 |
| 21 | 0.9 | 1 | 2.2 | 2 | 1 |  | IA | IA |  | 0 | 0 |
| 22 | 0.9 | 0.9 | 1.6 | 2 | 1 |  | IA | IIA |  | 0 | 3.9 |
| 23 | 1.2 | 1.6 | 1.7 | 1 | 1 |  | IA | IIA |  | 0 | 7.6 |
| 24 | 0.9 | 1.2 | 3.7 | 2 | 1 |  | IA | IA |  | 0 | 0 |
| 25 | 1.6 | 1.4 | 1.3 | 1 | 1 |  | IA | IIA |  | 0 | 4.5 |
| 26 | 1 | 1.6 | 2.2 | 2 | 1 |  | IIA | IIA |  | 13.5 | 13.3 |
| 27 | 0.9 | 0.9 | 2.1 | 2 | 1 |  | IA | IA |  | 0 | 0 |
| 28 | 0.9 | 1 | 2.4 | 2 | 1 |  | IIB | IA |  | 12.7 | 0 |
| 29 | 1.4 | 1.4 | 1.7 | 2 | 2 |  | IA | IA |  | 0 | 0 |
| 30 | 1 | 1 | 3 | 1 | 1 |  | IIA | IIA |  | 2.4 | 2.4 |
| 31 | 0.9 | 0.9 | 3.3 | 2 | 1 |  | IA | IA |  | 0 | 0 |
| 32 | 1.3 | 1.2 | 1.6 | 1 | 1 |  | IIA | IA |  | 13.8 | 0 |
| 33 | 0.9 | 0.9 | 2.9 | 2 | 1 |  | IA | IA |  | 0 | 0 |
| 34 | 1.4 | 1 | 1.5 | 1 | 1 |  | IIA | IIA |  | 10.2 | 10.4 |
| 35 | 1.1 | 0.9 | 2.8 | 1 | 1 |  | IIB | IA |  | 5 | 0 |
| 36 | 0.9 | 0.9 | 1.3 | 2 | 2 |  | IIA | IA |  | 7.4 | 0 |
| 37 | 0.9 | 1 | 5 | 1 | 1 |  | IIA | IA |  | 3.6 | 0 |
| 38 | 1 | 1 | 2 | 1 | 1 |  | IA | IIA |  | 0 | 4.2 |
| 39 | 1 | 1 | 1.3 | 1 | 2 |  | IIA | IA |  | 15.9 | 0 |
| 40 | 1.1 | 1.2 | 1.3 | 1 | 2 |  | IIA | IIB |  | 5.1 | 13.5 |
| 41 | 1.1 | 1 | 1.5 | 2 | 1 |  | IA | IA |  | 0 | 0 |
| 42 | 1.1 | 1.1 | 1.3 | 2 | 1 |  | IA | IIA |  | 0 | 3.2 |
| 43 | 0.9 | 0.9 | 1.3 | 2 | 1 |  | IA | IA |  | 0 | 0 |
| 44 | 0.9 | 1 | 2.4 | 1 | 1 |  | IA | IA |  | 0 | 0 |
| 45 | 1.1 | 1 | 1.6 | 1 | 2 |  | IIA | IB |  | 5.5 | 3.3 |
| 46 | 1 | 0.9 | 3.2 | 1 | 2 |  | IA | IIB |  | 0 | 4.3 |
| 47 | 0.9 | 1.3 | 2.1 | 1 | 1 |  | IA | IIA |  | 0 | 5.8 |
| 48 | 0.9 | 0.9 | 1.3 | 1 | 2 |  | IA | IIB |  | 0 | 4.1 |
| 49 | 0.9 | 0.9 | 1.8 | 2 | 2 |  | IA | IA |  | 0 | 0 |
| 50 | 1.2 | 1.3 | 1.4 | 1 | 1 |  | IA | IIB |  | 0 | 4 |
| 51 | 1 | 1.1 | 1.4 | 2 | 1 |  | IA | IA |  | 0 | 0 |
| 52 | 1.1 | 1.3 | 1.3 | 1 | 2 |  | IA | IA |  | 0 | 0 |
| 53 | 1.2 | 1.1 | 1.3 | 1 | 1 |  | IA | IA |  | 0 | 0 |
| 54 | 1.3 | 1 | 2 | 1 | 2 |  | IA | IA |  | 0 | 0 |
| 55 | 0.9 | 1.1 | 2.3 | 2 | 1 |  | IA | IA |  | 0 | 0 |
| 56 | 1.1 | 0.9 | 3.6 | 1 | 2 |  | IA | IA |  | 0 | 0 |
| 57 | 1 | 0.9 | 1.3 | 1 | 2 |  | IA | IA |  | 0 | 0 |
| 58 | 1 | 1.1 | 2.3 | 2 | 1 |  | IIA | IA |  | 3.6 | 0 |
| 59 | 1.1 | 0.9 | 4.8 | 1 | 1 |  | IA | IIA |  | 0 | 4 |
| 60 | 1.3 | 0.9 | 2.3 | 2 | 1 |  | IIB | IA |  | 4 | 0 |

L, Left; R, Right; ASV, Anterior septal vein; SP, Septal point; ICV, Internal cerebral vein; FM= foramen of Monro.
